# Increasing Nitrogen Use Efficiency of Corn in Midwestern Cropping Systems

**DOI:** 10.1100/tsw.2001.352

**Published:** 2001-11-21

**Authors:** J.L. Hatfield, J.H. Prueger

**Affiliations:** USDA-Agricultural REsearch Service, National Soil Tilth Laboratory, Ames, IA 50011, USA

**Keywords:** water use, water use efficiency, corn yield, drainage, water quality, nitrogen management

## Abstract

Nitrogen (N) loss from agricultural systems raises concerns about the potential impact of farming practices on environmental quality. N is a critical input to agricultural production. However, there is little understanding of the interactions among crop water use, N application rates, and soil types. This study was designed to quantify these interactions in corn (Zea mays L.) grown in production-size fields in central Iowa on the Clarion-Nicollet-Webster soil association. Seasonal water use varied by soil type and N application rate. Yield varied with N application rate, with the highest average yield obtained at 100 kg ha. N use efficiency (NUE) decreased with increasing N application rates, having values around 50%. Water use efficiency (WUE) decreased as N fertilizer rates increased. Analysis of plant growth patterns showed that in the low organic matter soils (lower water-holding capacities), potential yield was not achieved because of water deficits during the grain-filling period. Using precipitation data coupled with daily water use throughout the season, lower organic matter soils showed these soils began to drain earlier in the spring and continued to drain more water throughout the season. The low NUE in these soils together with increased drainage lead to greater N loss from these soils. Improved management decisions have shown that it is possible to couple water use patterns with N application to increase both WUE and NUE.

## INTRODUCTION

The role of agricultural practices and their impact on environmental quality have been carefully examined in the past 2 decades because of the suggestion of a relationship between decreasing environmental quality and increasing agricultural production. The recurrence of the hypoxic zone in the Gulf of Mexico and the substantial increase in the size of the hypoxic zone after the 1993 floods in the Midwest focused attention on the role of agriculture in nonpoint source pollution. Burkart and James[1] evaluated the nitrogen (N) balance for the Mississippi River Basin and concluded that mineralization of soil organic matter and application of commercial fertilizer were two primary contributors to N load. Jaynes et al.[2], after examination of a small watershed (5400 ha) in central Iowa, found that nitrate-N losses averaged 20 kg ha$^{-1}$ and reached a level in excess of 40 kg ha$^{-1}$ during 1993. Hatfield et al.[3] found that drainage from Walnut Creek was the primary transport pathway for nitrates and that annual loads were related to precipitation differences among years. In the Walnut Creek watershed, drainage through the subsurface drain lines accounted for approximately half of the annual precipitation, with evapotranspiration accounting for the other half (Hatfield et al.[4]). This movement of water through the soil profile together with the solubility of nitrate in water produce large amounts of nitrate-N loss (Jaynes et al.[2]). These results suggest a strong link among precipitation, crop water use patterns, and nitrate losses. Occurrence of the hypoxic zone has prompted an increased level of debate about the need to reduce N inputs into agricultural systems. Conversations with crop producers in the Midwest reveal their concerns that over the past 20 years, their input of N fertilizer has not increased, soil organic matter levels have not changed, and crop production levels have increased, suggesting that the efficiency of the agronomic production system has increased and any change in nitrate loss would be difficult to achieve.

Keeney and DeLuca[5] showed that the N concentrations in the nearby Des Moines River have not changed between the 1940s and the 1980s. They attributed this to the release of soil organic matter by cultivation in the mid-1940s and by the use of commercial fertilizers in the 1980s. There has been a change in this trend over the past 5 years, suggesting substantial changes in the N dynamics within the Raccoon River watershed. Anderson et al.[6] showed how land management practices for N affected groundwater quality in Minnesota. Schilling and Libra[7] demonstrated that intensity of land use in watersheds was directly related to nitrate-N concentrations in surface water of several Iowa watersheds. However, Hatfield et al.[8] found a weak relationship in the nitrate-N concentrations in surface water of midwestern watersheds for the harvested area and the fraction of harvested land to total area. There was not a significant relationship between the amount of N fertilizer applied and the nitrate-N concentrations in midwestern rivers.

Spatial variation of crop yield across fields has prompted a series of questions about the role of nutrient management. Jaynes and Colvin[9] showed that yield variation within central Iowa fields was related to precipitation differences in various years. Hatfield and Prueger[10] found a large amount of spatial variation in water use across fields related to soil types and N management. These results have prompted a series of studies developed to further elucidate these interactions. Studies conducted on water use and N application have been done within a single soil type or management zone. There is a lack of information on interactions among soil, crop water use, and N management that would help develop an understanding of improved N management for cropping systems typical of the Midwest. The increasing concern about the role of agricultural practices on water quality has prompted us to ask a series of questions about the interactions between crop water and N use, yield, and soils. The objectives of our studies have been twofold: first, to quantify the interactions among N application, crop water use, yield, and soils for central Iowa production fields on the Clarion-Nicollet-Webster soil association and, second, to determine the impact of these interactions on N management and potential offsite impacts from drained agricultural lands.

## EXPERIMENTAL METHODS AND PROCEDURES

Studies have been conducted in the Clarion-Nicollet-Webster soil association in central Iowa within the Walnut Creek watershed (Hatfield et al.[4]). This 5400-ha watershed is described in Hatfield et al.[4] Production-sized fields have been used as experimental units for these studies because of the need to quantify the affects of N rate application on crop yield and water use across a series of soil types within the Clarion-Nicollet-Webster soil association. These production fields have ranged in size from 32 to 96 ha. In each case, the experimental design has been similar. N rates applied in 1997 and 1998 used a starter application only at planting of 50 kg ha$^{-1}$. The second treatment had the starter rate (50 kg ha$^{-1}$) and a sidedress rate (applied at a growth stage of six to eight leaves) determined by the use of the Late Spring Nitrate Test. The third treatment was the starter plus a sidedress rate to represent a nonlimiting N rate of an additional 150 kg ha$^{-1}$. This experimental procedure was described by Jaynes et al.[11] In 1999 and 2000, the N application procedure was modified to further refine N application rates based on leaf chlorophyll response and soil test obtained from the 1997 and 1998 experiments. The rates applied were 50, 100, and 150 kg ha$^{-1}$ to a series of different soils, planting rates, and plant population densities (75,000 and 85,000 plants ha$^{-1}$). N rates were applied uniformly across each field using liquid urea (UAN, 32% N) for these studies. Within a growing season, only a single field was studied, with changing agronomic inputs made within the field. Soil tests for pH, phosphorus, and potassium levels of the soils were measured in the fall after harvest, with phosphorus and potassium added to ensure adequate amounts for optimal corn production.

Soil N concentrations were measured prior to spring operations, after planting, and at the end of the growing season after harvest to a soil depth of 1.5 m using a 5-cm core. Cores were subdivided into depth increments to estimate the N availability throughout the root zone. Sample position was recorded with a GPS unit to ensure accurate location of each subsequent sample. Nominal plot size for plant measurements and yield determinations was 15 × 15 m. Yields were measured on 5-m length of row for five subsamples within each plot in which no plants had been removed or measured during the growing season. Plots were replicated three times within each treatment.

Crop water use rate was measured from planting to harvest using an energy balance method (Bowen ratio in 1997 and 1998, and eddy correlation in 1999 and 2000) to quantify daily evaporation totals. Use of these approaches for estimation of crop water use is described in Hatfield[12]. These units consisted of a net radiometer positioned at 3 m above the canopy, soil heat flux at 10 cm below the soil surface (within the row, middle of the row, and adjacent to the row), wind speed, air temperature, water vapor pressure (positioned at 0.5 and 1.5 m above the canopy), three-dimensional sonic anemometer and krypton hygrometer (positioned at 1 m above the canopy), and an infrared thermometer (15° fov) positioned at 45° from nadir in a south-facing direction. All measurements were recorded at 10-s intervals and either 15- or 30-min averages stored in the data acquisition unit. Data were screened to ensure proper data quality, converted to equivalent water depth, and summed for the growing season to determine crop water use. Within each soil-N combination, we had a single energy balance station, and this measurement technique represented an area of approximately 50 m$^2$. Surface soil water measurements of the upper 5 cm were made using gravimetric samples in each soil type for 1998 and a dielectric probe in 1999 and 2000. These data were collected twice weekly. These instruments were positioned to provide a water use estimate representative of the area of the plant measurements. Missing data for a given station were interpolated through the development of relationships within a soil type across N management practices among the energy balance variables. When data were missing, these relationships were used as the guide to fill in periods of less than 2 h. Generally, the period of missing data was less than 3 h. Missing or erroneous data often occurred at night, when evaporation rates are at a minimum. The amount of missing data for these experiments was less than 3% of the total data record.

Crop transpiration rates were estimated from a Penman-Monteith energy balance model that determined the soil water evaporation rate based on the leaf area index of the crop and previous precipitation amounts (Ritchie and Burnett[13]). Soil water evaporation rates were estimated from a Penman-Monteith surface energy balance model based on crop residue cover amounts and the energy balance. Precipitation amounts for these studies were available from a tipping bucket rain gauge located at a meteorological station within 1 km of the field sites. Total amounts of precipitation for the 4 years of this study represented a range of conditions that would be typical of the variation encountered during a number of growing seasons.

Crop growth and development were measured in a variety of ways. In 1997, measurements of yields were made at harvest. In 1998 and 1999, a more intensive regime of weekly plant measurements (consisting of leaf area, phenological stage, number of leaves, dry weight, and plant height) were made on ten plants from each plot replicated three times. In 2000, the frequency was decreased to four destructive plant samplings to represent the 6-leaf stage, 12-leaf stage, tasseling, and mid-grain fill. Leaf chlorophyll measurements were made on 30 plants in each plot with a leaf chlorophyll meter twice per week, commencing with the 6-leaf stage and continuing through late grain fill. The upper leaf was measured at the mid-leaf position until the tassel appeared, after which the leaf immediately above the ear position was measured. Leaf carbon and N contents were determined on dry, ground samples from the same stages as the 2000 plant samples were collected. For the 1999 and 2000 experiments, stalk sugar content was measured on ten plants from each plot with a sugar refractive method using sap collected from freshly cut stalks. Grain quality parameters of protein, oil, and starch were measured on subsamples of grain collected from the hand-harvest samples. Field yields were measured with yield monitors mounted on the producers' combine. These data were registered with a GPS unit to obtain accurate field locations.

Data analyses for these studies were based on crop yield, total seasonal water use, and N application. Water use efficiency (WUE) and N use efficiency (NUE) were determined by the ratio of crop yield to either seasonal crop transpiration or N application rates. Observation points for plant parameters shown in this report are the mean of the three replicates of each N treatment within a soil type. Water use measurements represent a single value for each N treatment-soil type combination; however, the values represent an area similar in size to the plant sampling area. Intensive plant sampling data are not described in this report but were used to understand the dynamics of plant response to changes within season N management decisions. Likewise, leaf chlorophyll and stalk sugar content data were used to guide decisions in the 1999 and 2000 experiments. These data sets represent a complete analysis of crop-soil-water-N interactions.

## RESULTS AND DISCUSSION

### Crop Yield-N Relationships

Corn yield exhibited a large increase as N application rates increased above 50 kg ha$^{-1}$ in 1997 (Fig. 1). The more interesting observations were in the variations among the soils in this study. At the 50 and 150 kg ha$^{-1}$ rates, there was little variation among the Clarion, Okoboji, and Webster soils. However, at the 200 kg ha$^{-1}$ rate, yield differences among soils increased considerably (Fig. 1).

In 1998, corn yields from Canisteo, Clarion, and Nicollet soils across three N rates showed a significant interaction of soil and N rate for grain yields (Fig. 2). These differences can be

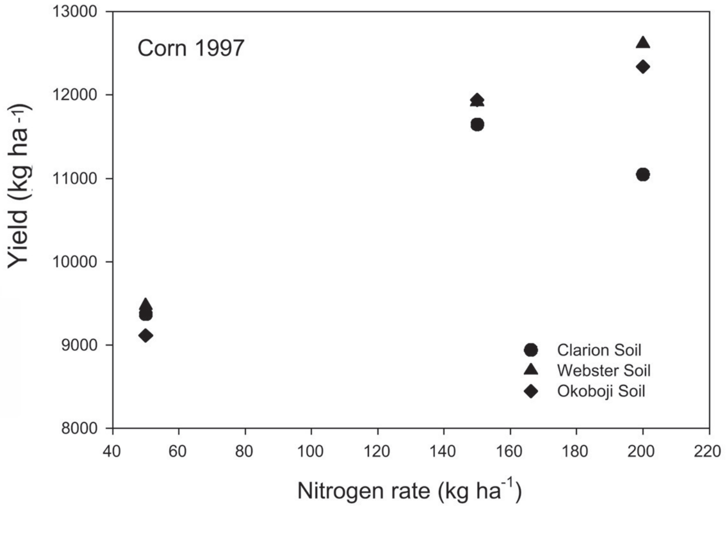

**FIGURE 1**. Yield of corn in three different soils at three N application rates for 1997.

attributed to precipitation amounts during the growing season, with the June-July period in 1998 receiving over 300% of normal precipitation. Yields were lower than in 1997, and the Clarion soil had the highest yields (Fig. 2). In 1999 and 2000 for the different soil-N combinations, the observed yield differences among soils began to separate when N application rates increased above 100 kg $ha^{-1}$. These observations across 4 years suggest a strong interaction between N rate and soil type.

Combining all of the observed yield data across soils and N application rates for the 1998–2000 period revealed a large amount of variation in the yields for a given application rate (Fig. 3). Fitting a linear regression line for the 50 to 100 kg $ha^{-1}$ rate showed there was a 70 kg $ha^{-1}$ increase in yield for each unit of N applied. Similarly, when a line was fit through the 100 to 200 kg $ha^{-1}$ rates, yields decreased 4.5 kg $ha^{-1}$ for each unit of N. When the data were segregated by soils, the lower organic matter soils were clustered in the lower yields within a given N application rate, whereas the higher organic matter soils were associated with higher yields for a given N rate.

## Crop Water Use Patterns

Observed crop water use rates measured with energy balance methods showed a large amount of scatter across soils and N rates (Fig. 4). Within each soil, a large amount of variation was associated with each change in N rate. Across years, seasonal precipitation ranged from 372 mm in 2000 to 744 mm in 1998. However, when we examined the relationship among precipitation, crop transpiration, and N rates, there was a large amount of variation within N rates with no definite pattern. The highest water use rates were from the 200 kg $ha^{-1}$ in the Canisteo and Clarion soils from the 1998 season. These data were obtained in a season with excessive precipitation, and a considerable portion of the seasonal water use can be attributed to evaporation from standing water on the soil surface. We found the 200 kg $ha^{-1}$ N rate had the highest water use across all years even though there was

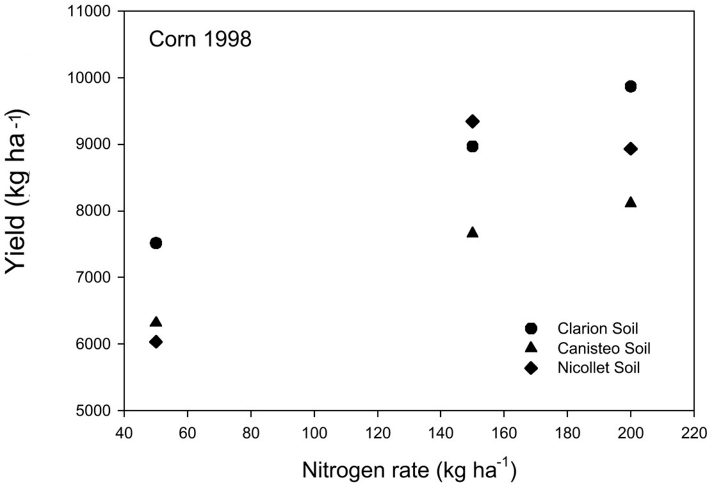

**FIGURE 2**. Yield of corn in three soils at three N application rates from 1998.

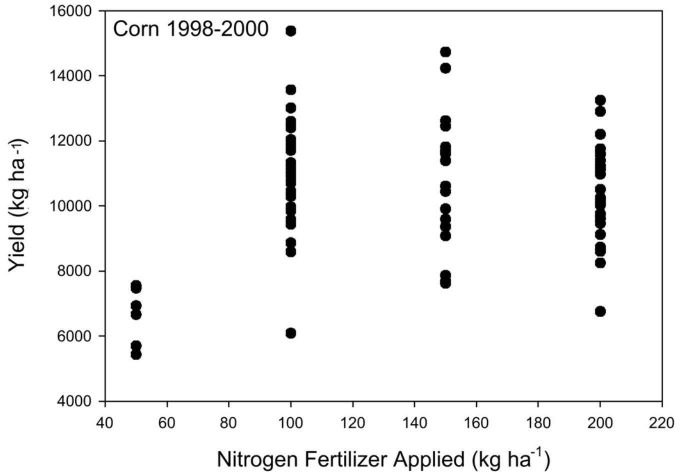

**FIGURE 3**. Corn yields observed for 1997–2000 experiments with four N application rates.

a twofold difference in precipitation among years. Water use variations among the soils and N rates suggest that differences in yield response are associated with these interactions. However, there is little information directly comparing crop water use to N management within a single field.

## Crop WUE

WUE was calculated from the transpiration losses from each N rate-soil combination relative to the crop grain yield. Transpiration losses account for only the amount of water evaporated from the plant surfaces during the growing season. In extremely wet years such as 1998, the soil water evaporation component could be quite large and produce an unrealistic assessment of crop water use. In this analysis, we assumed that the 150 kg ha$^{-1}$ N rate represents the optimal rate, as this is the one most used by producers. The line shown in Fig. 5 represents these data. For each plot, we calculated the WUE based on the mean of three yield observations from each plot coupled with an observation of crop water use. There was a large amount of scatter in these data for the 1997–2000 data. The fit to the observed values between yield and transpiration losses represents the WUE value. The slope of the line derived from these observations and shown in Fig. 5 is typical for midwestern cropping systems (Hatfield et al.[14]). More interesting is the observation that most of the yield-transpiration values for many of the plots were below the regression line, suggesting that N management practices are not creating an optimal use of water relative to crop yield. Hatfield et al.[14] found in reviewing the literature that there were several studies on the effect of N management on WUE; however, there was no information on variation across soils within the same study and no detailed comparison of water use within fields for a range of N management practices. Comparisons of crop water use have often been directed toward management effects across different species or across years, but no data exist for this type of detail within a single field. There were a few yield observations above the regression line that were associated with a 100 kg ha$^{-1}$ N rate as a result of improved management practices instituted in 2000.

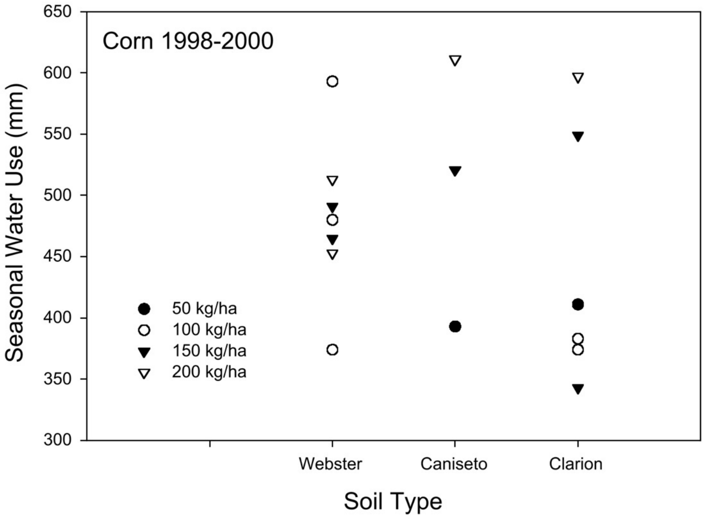

**FIGURE 4**. Seasonal water use from corn grown in different soil types with four different N application rates in central Iowa.

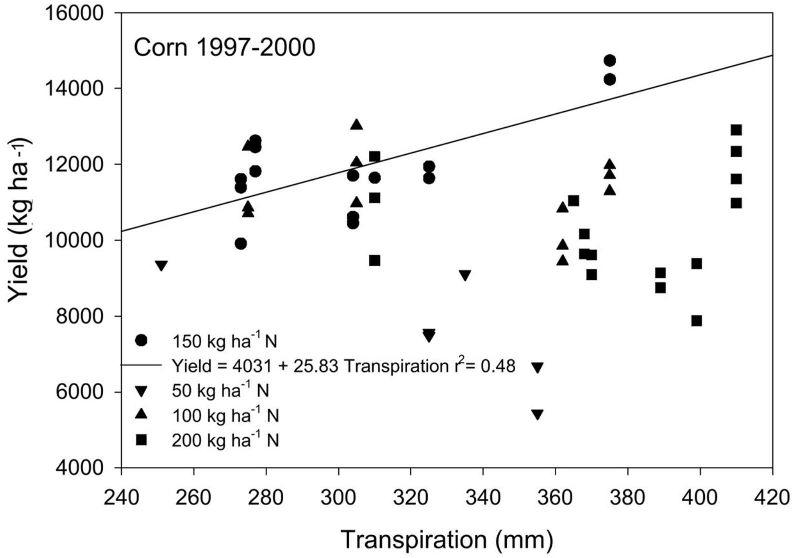

**FIGURE 5**. Crop yield-transpiration relationships developed for corn grown in various soil-N application rate combinations for the 1997–2000 period. Line fit through the 150 kg ha$^{-1}$ observations.

These data suggest that it is possible to alter WUE by N management and that linking our understanding of water use patterns to N management across soils would enhance crop response.

## NUE

Another expression of crop response to N is the determination of NUE. This type of expression leads to efficiencies above 100% when N mineralization in the soil provides a significant portion of the N needs of the crop. NUE decreased as the amount of N applied increased (Fig. 6). The relationship between N applied and NUE was linear for this study. These data were based on N application rates and not on N utilization from the soil profile. Data are available from 1999 and 2000 to determine the N uptake based on N concentrations in the plant biomass and grain.

An interesting observation in these data is the variation around the line in Fig. 6. The variation in NUE was less than for the yield-N relationship (Fig. 3). In these data, the lower organic matter soils represented the lower NUE values for a given N application rate, and the higher organic matter soils represented the upper range of NUE.

Corn yields in response to N application rates behaved in the pattern accepted by agronomists (Fig. 3). However, the optimal rate is much lower than the normal application rate of 150 kg $ha^{-1}$. The 4 years of this study had a wide range of precipitation totals and represent the typical range of conditions experienced in central Iowa. WUE values showed a decline when N application rates were over 150 kg $ha^{-1}$; however, many of the lower N application rates, e.g., 50 kg $ha^{-1}$, also exhibited low WUE values. Throughout each of the growing seasons, an interesting pattern began to emerge for crop water use and crop growth patterns. In central Iowa, the precipitation pattern is such that recharge of the soil profile occurs by late March or early April. Beginning in late June, the transpiration rates exceed the precipitation amounts, at which point the crop begins to extract water stored in the soil profile. We found in the growth analysis of the crop across these years that the higher rates of N applied increased the early-season plant growth as well as the potential yield of the crop. The early management decisions allowed the plant to develop an amount of biomass capable of producing a large crop. However, in this precipitation regime, we found that during the grain-filling period, water deficits were often encountered that reduced the ability of the plant to achieve this yield potential. This was evident in the Clarion and Canisteo soils that are the lower organic matter soils with diminished water-holding capacities. We observed that in these soils, the potential yield at the onset of grain-filling was nearly equal to the other soils within the fields, but the harvested yields were reduced as much as 25%. The only exception to this was the extremely wet summer of 1998, when there was an advantage to the better-drained soils (i.e., low organic matter). These results show that N management relative to soil type has to account for soil water-holding capacity, seasonal water use, and precipitation patterns.

In 2000, we modified N management for this field to begin to account for water use and precipitation amounts. Using leaf chlorophyll measurements to determine N status of the plant and water use rates, we determined midway through the vegetative growth period that an adequate amount of N was available for optimal crop yield. These decisions produced the higher WUE and yields shown in Fig. 5. N management can produce increased WUE and across fields contribute to increased spatial variation in yield. Observations of residual soil N after harvest from the 1999 and 2000 experiments showed that more N was left in the soil in the Clarion and Canisteo soils compared to the Webster or Nicollet soils within this field.

## Soil Drainage Patterns

Observations of higher N residual amounts in the soil profile in the Clarion and Canisteo soils coupled with soil water use pat-

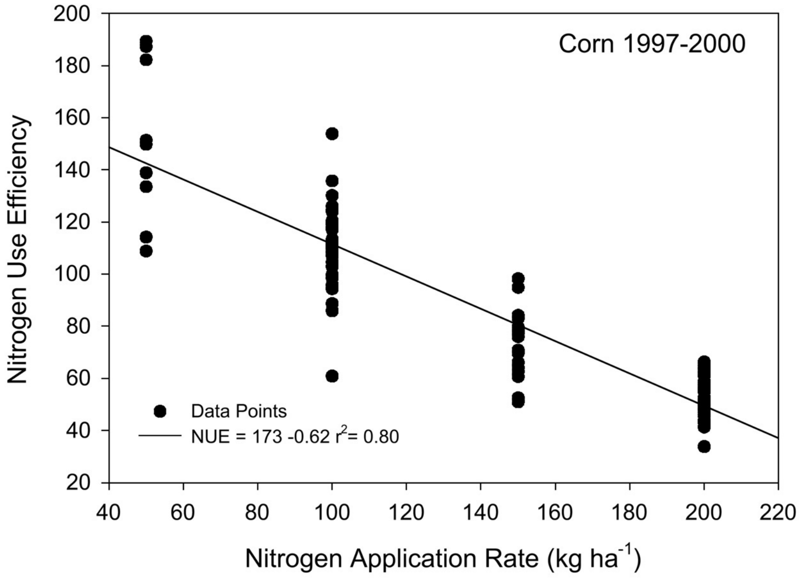

**FIGURE 6**. NUE for corn grown under different N application rates for central Iowa production fields in 1997-2000.

terns raised a question about the drainage dynamics in these soils. We developed a simple soil water balance model described in Hatfield et al.[8] as

$$\text{If } TSW_{(t-1)} + P_t - Et_t > TSW_{FMC},$$
$$\text{then } (TSW_{(t-1)} + P_t - Et_t) - TSW_{FMC} = D_t \quad (1)$$

where $TSW_{(t-1)}$ is the total amount of water stored in the 1.2-m soil profile on the previous day (t – 1), t is time in days, $P_t$ is precipitation, $Et_t$ is evapotranspiration from the field, $TSW_{FMC}$ is total stored soil water in the 1.2-m profile at water-holding capacity, and $D_t$ is drainage from the soil profile. The database used to populate this model was the observed precipitation, estimated Et from the Penman-Monteith model for the different soils within the field, and the soil water-holding capacity data measured from soil properties for each of these soil types within the Walnut Creek watershed. This equation was used to predict the amount of drainage from the different soils within the fields.

Predicted drainage patterns for the different years produced some interesting results. Drainage from the Clarion soil in 1997 under the 150 kg ha$^{-1}$ N rate began earlier and produced more cumulative drainage than from the Okoboji soil (Fig. 7). Drainage from the Clarion soils commenced 30 days before the Okoboji soils and also had drainage late in the year when the soil profile was recharged by late fall precipitation events. In 1998, there was a different pattern of drainage between the Clarion and Canisteo soils (Fig. 8). In that year, the Okoboji soils were not measured because of the small area within the field and the excess precipitation produced standing water that drowned the corn

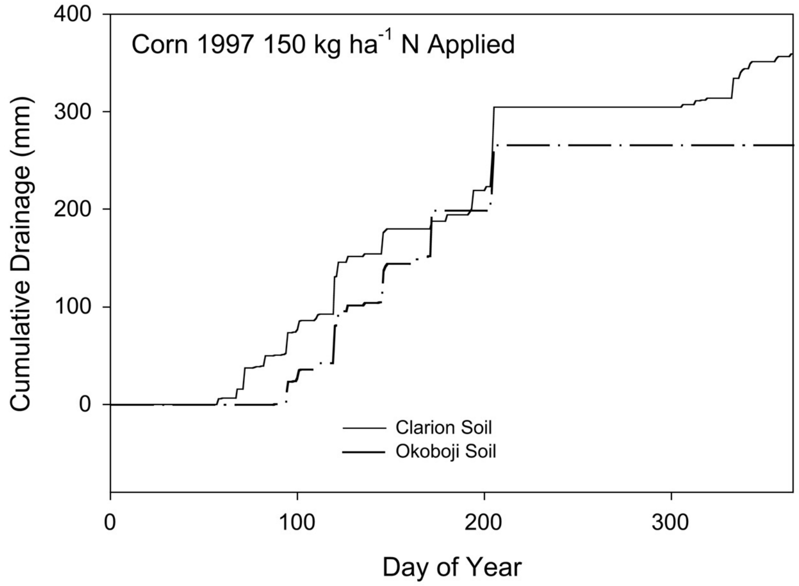

**FIGURE 7**. Cumulative drainage during 1997 from Clarion and Okoboji soils under the 150 kg ha$^{-1}$ N rate.

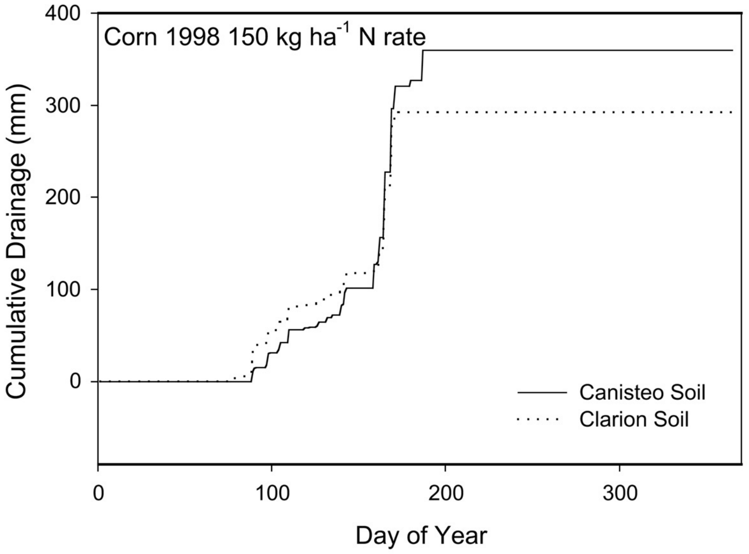

**FIGURE 8**. Cumulative drainage patterns of Canisteo and Clarion soils in 1998 at the 150 kg ha$^{-1}$ N rate.

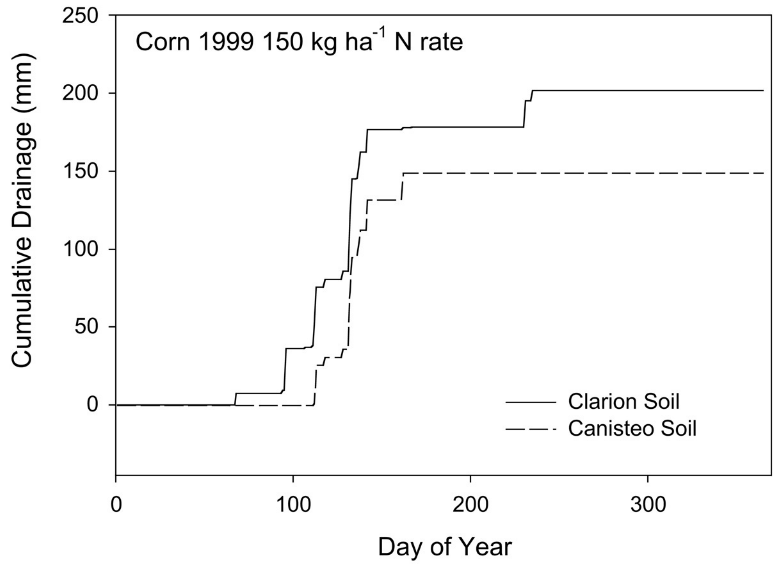

**FIGURE 9**. Cumulative drainage patterns of Canisteo and Clarion soils in 1999 at the 150 kg ha$^{-1}$ N rate.

plants. The Canisteo soils produced more drainage than the Clarion in 1998 because there was less crop growth on the Canisteo soils.

In 1999, the drainage pattern changed, with the Clarion soils beginning earlier and producing more drainage than the Canisteo soils (Fig. 9). There was a temporal difference of almost 40 days in the onset of drainage from the soil profile, and the Clarion soils showed some drainage from precipitation events late in the year.

These drainage patterns— coupled with the increase in residual N created by inefficient crop uptake due to late-season water deficits—create a situation in which residual N can move from the soil profile very quickly in the spring. Well-drained soils require a N management strategy that accounts for soil water-holding capacity and growing season precipitation to produce the highest WUE and NUE. We have been able to integrate these factors into management systems that have produced in excess of 14,000 kg ha$^{-1}$ of grain with 100 kg ha$^{-1}$ of applied N. Further refinement of these interrelated factors will produce even more gains in efficient agricultural systems.

## CONCLUSIONS

Water use patterns by corn and N management interact depending upon the soil type and the seasonal precipitation. In these studies, conducted across a range of soil types within the same field, it was found that grain yield varied with N rate; however, there was a large amount of yield variability (±50%) within a given N rate. Water use rates varied with N application rate and soil type by as much as 300 mm within a growing season, and the difference in growing-season precipitation was not the dominant factor in determining crop water use amounts. Seasonal water use rates were increased by N application because increased N enhanced early-season crop growth and thus water use. In water-limited soils (Clarion and Canisteo), this reduced the yield due to low available soil water during the grain-filling period. Drainage totals from the different soils showed that low organic matter soils began to drain earlier in the spring and had more cumulative drainage than the higher organic matter soils in the landscape. NUE can be increased in corn production when coupled with an understanding of crop water use patterns. The net result is an improvement in the efficiency of nutrient use and a reduction of environmental impact. Extension of these results to the Midwest will require an integration of N management practices and intensity of subsurface drainage linked to crop water use patterns to improve both production efficiency and environmental quality.

## ACKNOWLEDGMENTS

This study would not be possible without the dedication and efforts of Tim Hart and Wolfgang Oesterreich and their diligence in the supervision of the summer students.

| | | |
|---|---|---|
| Received: | July | 17, 2001 |
| Revised: | October | 10, 2001 |
| Accepted: | November | 1, 2001 |
| Published: | November | 21, 2001 |

## BIOSKETCH

**Jerry L. Hatfield** is Laboratory Director of the USDA-ARS National Soil Tilth Laboratory in Ames, Iowa. He received his Ph.D. degree from Iowa State University in 1975 in Agricultural Meteorology and his research interests are in the quantifying the energy exchanges in cropping systems to determine the environment impact of different management systems. His current research focus is on the integration of water, nitrogen, light, and carbon dynamics in corn-soybean production systems. He is the co-editor of the book *Nitrogen in the Environment: Sources, Problems, and Management* along with Ron Follett.
